# Typ-D-Persönlichkeitsstil und Rückenschmerzen: Eine narrative Übersicht zur Relevanz psychosozialer Faktoren in der orthopädischen Schmerztherapie

**DOI:** 10.1007/s00132-025-04718-4

**Published:** 2025-09-09

**Authors:** Christian Riediger, Mark Ferl, Christoph H. Lohmann, Maria Schönrogge

**Affiliations:** Orthopädische Universitätsklinik Magdeburg, Medizinische Fakultät, Otto-von-Guericke-Universität Magdeburg, Magdeburg, Deutschland

**Keywords:** Psychosomatische Störungen, Chronischer Schmerz, Multimodale Therapie, Risikofaktoren, Soziale Unterstützung, Psychosomatic disorders, Chronic pain, Multimodal treatment, Risk factors, Social support

## Abstract

**Hintergrund:**

Die Typ-D-Persönlichkeit („distressed personality“) ist charakterisiert durch die Kombination negativer Affektivität und sozialer Inhibition. Während dieser Persönlichkeitsstil ursprünglich im Kontext kardiovaskulärer Erkrankungen erforscht wurde, belegen aktuelle Studien auch eine signifikante Assoziation mit chronischen Schmerzerkrankungen, insbesondere Rückenschmerzen.

**Fragestellung:**

Diese narrative Übersicht untersucht den aktuellen Kenntnisstand zur Beziehung zwischen Typ-D-Persönlichkeit und Rückenschmerz. Ziel ist es, mögliche psychologische, verhaltensbezogene und biologische Mechanismen zu analysieren und klinische Implikationen für die orthopädische Schmerztherapie abzuleiten.

**Material und Methode:**

Es wurde eine selektive Literaturrecherche in den Datenbanken PubMed, PsycINFO und Google Scholar durchgeführt. Berücksichtigt wurden Originalarbeiten, systematische Reviews und Metaanalysen der letzten 20 Jahre mit Fokus auf psychosomatische Aspekte chronischer Rückenschmerzen.

**Ergebnisse:**

Mehrere Studien zeigen, dass Personen mit Typ-D-Merkmalen ein erhöhtes Risiko für das Auftreten und die Chronifizierung von Rückenschmerzen aufweisen. Mögliche vermittelnde Mechanismen sind maladaptive Stressverarbeitung, Somatisierung, reduzierte soziale Unterstützung sowie Veränderungen in neuroendokrinen und inflammatorischen Prozessen.

**Schlussfolgerungen:**

Die Typ-D-Persönlichkeit ist ein relevanter psychosozialer Risikofaktor in der Entstehung und Persistenz chronischer Rückenschmerzen. Ihre frühzeitige Erkennung könnte zur Verbesserung multidisziplinärer Therapieansätze beitragen.

## Abstract (en)

**Background:**

The Type D personality (“distressed personality”) is characterized by a combination of negative affectivity and social inhibition. While this personality style was originally researched in the context of cardiovascular disease, recent studies also show a significant association with chronic pain disorders, especially back pain.

**Objectives:**

This narrative review examines the current state of knowledge on the relationship between type D personality and back pain. The aim is to analyze possible psychological, behavioral, and biological mechanisms and to derive clinical implications for orthopedic pain therapy.

**Materials and methods:**

A selective literature search was conducted in the PubMed, PsycINFO and Google Scholar databases. Original studies, systematic reviews and meta-analyses from the last 20 years focusing on psychosomatic aspects of chronic back pain were included.

**Results:**

Several studies show that individuals with Type D characteristics have an increased risk of developing and chronicizing back pain. Possible mediating mechanisms include maladaptive stress processing, somatisation, reduced social support and changes in neuroendocrine and inflammatory processes.

**Conclusions:**

Type D personality is a relevant psychosocial risk factor in the development and persistence of chronic back pain. Early detection may contribute to improving multidisciplinary therapeutic approaches.

## Einleitung

Chronische Rückenschmerzen zählen zu den häufigsten muskuloskelettalen Beschwerden in Deutschland und stellen eine erhebliche sozioökonomische Belastung dar. Obwohl strukturelle Ursachen häufig fehlen oder nicht korrelieren mit der Schmerzintensität, wird in der aktuellen Literatur zunehmend die Bedeutung psychosozialer Einflussfaktoren betont [6]. Das biopsychosoziale Modell der Schmerzchronifizierung bietet einen integrativen Erklärungsansatz für diese multidimensionale Problematik [10, 14, 20].

Die Typ-D-Persönlichkeitsstruktur, ein relativ junges Konstrukt der Persönlichkeitspsychologie, wurde zunächst im Bereich der psychosomatischen Kardiologie beschrieben [5]. Inzwischen mehren sich Hinweise, dass Typ D auch in der Schmerzmedizin eine prognostisch relevante Rolle spielt, insbesondere im Hinblick auf Rückenschmerzen [3, 8, 22].

## Typ-D-Persönlichkeit: Konzept und Diagnostik

Die Typ-D-Persönlichkeit setzt sich aus zwei stabilen Persönlichkeitsdimensionen zusammen:

- *Negative Affektivität (NA):* Tendenz, häufig negative Emotionen wie Ärger, Angst oder Reizbarkeit zu erleben.
- *Soziale Inhibition (SI):* Neigung, emotionale und soziale Zurückhaltung zu zeigen, insbesondere aus Angst vor Zurückweisung oder negativer Bewertung.

Erfasst wird Typ D standardisiert über den DS14-Fragebogen von Denollet [2, 4]. Ein Summenscore ≥10 in beiden Subskalen definiert eine Typ-D-Ausprägung. Personen mit Typ-D-Merkmalen neigen zu einem dysfunktionalen Stressverarbeitungsstil und einer verminderten sozialen Unterstützung [7].

## Rückenschmerz und psychosoziale Risikofaktoren

Psychosoziale Einflussgrößen wie Depression, Angst, Katastrophisierung und passive Coping-Strategien sind in der Ätiologie und Aufrechterhaltung chronischer Rückenschmerzen gut belegt [12]. Es ist zunehmend anerkannt, dass psychosoziale Faktoren nicht nur Folge, sondern auch Ursache und Verstärker chronischer Rückenschmerzen sein können – gemäß dem biopsychosozialen Modell. Die Integration dieser Faktoren in sogenannte „yellow flags“ hat Eingang in zahlreiche Leitlinien zur Rückenschmerzbehandlung gefunden.

Typ D kann als Persönlichkeitsmerkmal angesehen werden, das mehrere dieser Risikofaktoren in sich vereint [7]. Es stellt daher potenziell einen übergeordneten Prädiktor für ungünstige Schmerzverläufe dar [7, 18].

## Aktuelle Evidenzlage zur Assoziation von Typ D und Rückenschmerz

### Methodik

#### Einschlusskriterien

Eingeschlossen wurden Originalarbeiten, systematische Reviews oder Metaanalysen, die einen Zusammenhang zwischen Typ-D-Persönlichkeit und Rückenschmerzen untersuchen. Die Studienpopulation umfasste Erwachsene ab 18 Jahren. Die Erhebung der Typ-D-Persönlichkeit musste mittels validierter Instrumente, wie dem DS14-Fragebogen, erfolgen. Zusätzlich mussten Schmerzparameter wie Schmerzintensität, Schmerzchronifizierung oder funktionelle Einschränkungen erhoben werden. Berücksichtigt wurden nur Veröffentlichungen in Peer-reviewten Fachzeitschriften in englischer oder deutscher Sprache.

#### Ausschlusskriterien

Ausgeschlossen wurden Fallberichte, Kommentare, Editorials oder Konferenz-Abstracts ohne vollständige Daten. Ebenfalls ausgeschlossen wurden Studien mit Fokus auf andere Schmerzlokalisationen ohne spezifische Auswertung für Rückenschmerzen sowie Populationen mit primären psychischen Erkrankungen ohne orthopädischen Schmerzbezug.

#### Zeitraum der Recherche

Die Literaturrecherche umfasste den Zeitraum von Januar 2005 bis Mai 2025.

#### Datenbanken und Suchstrategie

Die Recherche erfolgte in den Datenbanken PubMed, PsycINFO und Google Scholar. Es wurden folgende Suchbegriffe unter Verwendung Boolescher Operatoren kombiniert: („Type D personality“ OR „distressed personality“) AND („back pain“ OR „low back pain“ OR „chronic back pain“).

#### Stichworte

Typ-D-Persönlichkeit · Rückenschmerzen · Chronischer Schmerz · Psychosoziale Faktoren · Orthopädie · Schmerzpsychologie

#### Anzahl der berücksichtigten Arbeiten

Von insgesamt 97 identifizierten Publikationen erfüllten 8 Studien die Einschlusskriterien und wurden in die Analyse einbezogen.

In einer Vielzahl von Querschnittsstudien wurde ein signifikanter Zusammenhang zwischen Typ-D-Ausprägung und erhöhter Schmerzintensität, -dauer sowie -behinderung nachgewiesen [1, 13, 15, 16]. Auch Längsschnittuntersuchungen zeigen, dass Typ-D-Persönlichkeit die Chronifizierungswahrscheinlichkeit erhöht und mit schlechteren Therapie-Outcomes assoziiert ist [11].

Beispielhaft sei eine Metaanalyse von Williams et al. [21] genannt, in der Typ D mit einer mittleren Effektstärke (d ≈ 0,4–0,6) mit Schmerzintensität und funktioneller Einschränkung korreliert war.

### Mögliche Mechanismen der Assoziation

#### Psychologisch

Typ-D-Personen zeigen häufig maladaptive Kognitionen (z. B. Katastrophisieren), ausgeprägte Schmerzfokussierung und reduzierte Selbstwirksamkeit [19].

#### Verhaltensbezogen

Bewegungsvermeidung, geringes Gesundheitsverhalten und verzögerte Inanspruchnahme medizinischer Hilfe wirken chronifizierend [17].

#### Biologisch

Neuroendokrine Dysregulationen (z. B. Hyperaktivität der Hypothalamus-Hypophysen-Nebennieren-Achse) und eine erhöhte proinflammatorische Aktivität (z. B. Interleukin‑6, TNF-α) wurden bei Typ-D-Probanden vermehrt beobachtet [9].

### Kritische Bewertung der Studienqualität

Die Qualität der eingeschlossenen Studien variierte deutlich, sowohl in methodischer Stringenz als auch in der Kontrolle potenzieller Bias-Risiken. Ein Großteil der Arbeiten wies Stärken in der klaren Definition der Typ-D-Persönlichkeit sowie der Verwendung validierter Messinstrumente (z. B. DS14) auf. Dennoch bestanden relevante methodische Einschränkungen, die bei der Interpretation der Ergebnisse berücksichtigt werden müssen.

#### Studiendesign

Die Mehrheit der eingeschlossenen Untersuchungen folgte einem querschnittlichen Design. Dies erlaubt zwar die Analyse von Assoziationen zwischen Typ-D-Persönlichkeit und Rückenschmerzparametern, verhindert jedoch die Ableitung kausaler Schlussfolgerungen. Prospektive Kohorten- und Interventionsstudien waren in der Minderheit, zeigten jedoch eine höhere Evidenzstufe und erlaubten Aussagen zu zeitlichen Zusammenhängen.

#### Stichprobengröße und Repräsentativität

Während einige Studien über große und heterogene Stichproben verfügten (*n* > 500), basierten andere auf stark begrenzten Teilnehmerzahlen (< 100), was die statistische Power einschränkt und das Risiko zufälliger Befunde erhöht. Zudem waren mehrere Studien auf spezifische klinische Subgruppen beschränkt (z. B. Patient:innen in Rehabilitationskliniken), was die Generalisierbarkeit limitiert.

#### Messinstrumente und Outcome-Parameter

Die Erfassung der Typ-D-Persönlichkeit erfolgte in allen eingeschlossenen Arbeiten mittels validierter Instrumente, meist der DS14-Skala. Unterschiede in der Wahl und Operationalisierung der Schmerzparameter (z. B. NRS, VAS, ODI) erschwerten jedoch den direkten Vergleich der Ergebnisse. In einigen Fällen fehlten standardisierte Messzeitpunkte, was die Vergleichbarkeit weiter einschränkte.

#### Kontrolle von Störfaktoren

Ein Teil der Studien berücksichtigte wichtige Confounder wie Depression, Angststörungen oder soziodemographische Variablen in multivariaten Analysen. Andere Arbeiten verzichteten auf eine systematische Kontrolle, was das Risiko von Verzerrungen erhöht und die interne Validität einschränkt.

## Klinische Relevanz und therapeutische Konsequenzen

Für die orthopädische Praxis ergibt sich die Notwendigkeit, psychosoziale Risikoprofile – inklusive Typ-D-Persönlichkeitsmerkmale – frühzeitig zu identifizieren. In multimodalen Schmerzprogrammen könnten spezifische psychotherapeutische Module die Behandlungseffekte bei Typ-D-Patient:innen verbessern.

Die vorliegenden Ergebnisse verdeutlichen, dass die Typ-D-Persönlichkeit nicht nur ein psychosozialer Marker, sondern potenziell auch ein therapeutisch relevanter Modulator im Management von Rückenschmerzen ist. Daraus ergeben sich folgende praxisrelevante Konsequenzen:

- Früherkennung durch Screening.
  - Einsatz validierter Instrumente wie der Denollet Scale (DS14) in orthopädischen Ambulanzen und Rehabilitationszentren zur Identifikation psychosozial gefährdeter Patient:innen.
  - Positives Screening als Anlass für vertiefte psychosoziale Diagnostik.
- Integration in multimodale Therapieprogramme.
  - Patient:innen mit Typ-D-Merkmalen profitieren besonders von multimodalen Schmerzprogrammen mit psychotherapeutischen Elementen (z. B. kognitive Verhaltenstherapie, Akzeptanz- und Commitment-Therapie) kombiniert mit Physiotherapie.
  - Psychologische Interventionen gezielt auf Stressbewältigung, soziale Aktivierung und kognitive Umstrukturierung ausrichten.
- Anpassung ärztlicher Kommunikation.
  - Offenes Arzt-Patient-Gespräch aktiv fördern, da soziale Inhibition Rückfragen hemmen kann.
  - Regelmäßige, strukturierte Verlaufskontrollen zur Verbesserung der Adhärenz einplanen.
- Gezielte Schmerzaufklärung („pain education“).
  - Vermittlung der Zusammenhänge zwischen Stress, Emotionen und Schmerz zur Reduktion maladaptiver Krankheitsüberzeugungen.
  - Nutzung von Visualisierungen und Alltagssimulationen zur besseren Verständlichkeit.
- Vermeidung rein somatischer Therapiepfade.
  - Bei ausgeprägten Typ-D-Merkmalen sind rein interventionelle oder medikamentöse Ansätze oft unzureichend.
  - Psychosomatisch-orthopädische Co-Behandlung zur Reduktion des Chronifizierungsrisikos erwägen.
- Einbindung in Leitlinien und Versorgungsstrukturen.
  - Typ-D-Persönlichkeit als Risikofaktor in Leitlinien zur Behandlung chronischer Rückenschmerzen aufnehmen.
  - Systematische Implementierung in Rehabilitationskonzepte, Disease-Management-Programme und Berufsgenossenschaftsverfahren anstreben.

### Grenzen der bisherigen Forschung

Viele der vorliegenden Studien zur Typ-D-Persönlichkeit und Rückenschmerzen basieren auf querschnittlichen Designs sowie auf subjektiven Selbstberichten der Patient:innen. Dies erhöht das Risiko für Verzerrungen, insbesondere im Hinblick auf sozial erwünschtes Antwortverhalten und retrospektive Fehleinschätzungen („recall bias“). Obwohl zahlreiche Studien einen Zusammenhang zwischen der Typ-D-Persönlichkeit und Rückenschmerzen belegen, zeigen sich in der Literatur auch signifikante Konflikte und Inkonsistenzen, die eine differenzierte Betrachtung erfordern:

- Variabilität der Befunde zur Schmerzintensität und -chronifizierung.
  - Einige Studien berichten über einen klaren positiven Zusammenhang zwischen Typ D und erhöhter Schmerzintensität sowie Chronifizierung [1, 2], während andere keinen signifikanten Effekt oder nur schwache Korrelationen finden [15].
  - Diese Diskrepanz könnte durch Unterschiede in Studiendesign, Patientenauswahl oder verwendeten Messinstrumenten bedingt sein.
- Unterschiedliche Operationalisierung der Typ-D-Persönlichkeit.
  - Die verwendeten Erhebungsinstrumente und die Schwellenwerte zur Klassifikation variieren zwischen den Studien, was die Vergleichbarkeit der Ergebnisse erschwert.
  - Einige Arbeiten nutzen ausschließlich die Denollet Scale (DS14), andere modifizieren diese oder verwenden ergänzende psychometrische Verfahren.
- Heterogenität der Stichproben und Settings.
  - Studien reichen von kleinen klinischen Querschnittserhebungen bis hin zu groß angelegten Kohortenstudien.
  - Unterschiede in der Patientenpopulation (ambulant vs. stationär, akut vs. chronisch) sowie kulturelle und sozioökonomische Faktoren können die Befundlage beeinflussen.
- Mangel an Längsschnitt- und Interventionsstudien.
  - Viele Studien sind querschnittlich angelegt, wodurch kausale Zusammenhänge zwischen Typ D und Schmerzentwicklung nicht abschließend geklärt werden können.
  - Die wenigen vorhandenen Interventionsstudien zeigen zwar positive Effekte, sind jedoch häufig durch kleine Stichproben und kurze Nachbeobachtungszeiträume limitiert.

Darüber hinaus existieren unterschiedliche Definitionen und Messinstrumente zur Erfassung der Typ-D-Persönlichkeit, was die Vergleichbarkeit von Studien erschwert. Während der DS14-Fragebogen am weitesten verbreitet ist, bestehen weiterhin offene Fragen hinsichtlich seiner Sensitivität und Spezifität im klinischen Kontext.

Ein zentrales methodisches Defizit liegt zudem in der unzureichenden Klärung kausaler Zusammenhänge. Ob Typ-D-Merkmale Auslöser, Verstärker oder Folge chronischer Rückenschmerzen sind, ist bislang nicht abschließend geklärt.

Ein gezieltes Screening mittels kurzer Fragebögen wie DS14 ist praktikabel und sollte in interdisziplinären Settings berücksichtigt werden.

## Fazit

Für ein besseres Verständnis der Rolle der Typ-D-Persönlichkeit in der Schmerzmedizin sind folgende Forschungsansätze vorrangig:

- Durchführung *längsschnittlicher Studien*, um die kausale Wirkrichtung zwischen Persönlichkeitsmerkmalen und Schmerzverlauf zu klären.
- *Interventionsstudien*, die gezielt psychotherapeutische oder edukative Programme für Typ-D-Personen evaluieren, insbesondere in Kombination mit multimodalen Schmerztherapien.
- *Integration der Typ-D-Persönlichkeit* in bestehende Klassifikationssysteme und Behandlungsleitlinien für chronische Rückenschmerzen, um eine individualisierte Therapieplanung zu fördern.

Ein interdisziplinärer Ansatz – unter Einbezug von Psychologie, Orthopädie, Schmerztherapie und Gesundheitssoziologie – erscheint dabei besonders vielversprechend. Die Typ-D-Persönlichkeit stellt einen unabhängigen Prädiktor für die Chronifizierung und Therapieresistenz von Rückenschmerzen dar. Die Berücksichtigung dieses Konstrukts kann zur Entwicklung individualisierter, biopsychosozial fundierter Behandlungsansätze beitragen. Weitere Forschung, insbesondere in Form prospektiver Interventionsstudien, ist erforderlich.

## Abkürzungen

DS14: Denollet Scale 14, standardisierter Fragebogen zur Erfassung der Typ-D-Persönlichkeit
NA: Negative Affektivität, Persönlichkeitsdimension mit Tendenz zu häufigen negativen Emotionen
NRS: Numeric Rating Scale, numerische Ratingskala zur Schmerzmessung
ODI: Oswestry Disability Index, Fragebogen zur Erfassung funktioneller Einschränkungen bei Rückenschmerzen
SI: Soziale Inhibition, Persönlichkeitsdimension mit Neigung zu sozialer Zurückhaltung
TNF-α: Tumornekrosefaktor alpha, proinflammatorisches Zytokin
VAS: Visuelle Analogskala, Skala zur Messung der Schmerzintensität

## References

[1] Althoff R et al (2012) Type D personality and pain intensity in chronic low back pain. Eur J Pain 16(9):1229–1235

[2] Barnett MD, Ledoux T, Garcini LM et al (2009) Type D Personality and Chronic Pain: Construct and Concurrent Validity of the DS14. J Clin Psychol Med Settings 16:194–199. 10.1007/s10880-009-9152-0 19266270 10.1007/s10880-009-9152-0

[3] De Fruyt F, Denollet J (2002) Type D personality: a five-factor model perspective. Psychol Health 17(5):671–683

[4] Denollet J (2005) DS14: standard assessment of negative affectivity, social inhibition, and Type D personality. Psychosom Med 67(1):89–97 15673629 10.1097/01.psy.0000149256.81953.49

[5] Denollet J (1995) Personality and risk of cancer in men with coronary heart disease. Psychol Med 25(5):1063–1073 10.1017/s0033291797006442 9723154

[6] Eikeseth FF, Pedersen G, Hummelen B, Sütterlin S, Stubhaug A, Kvarstein EH, Kvarstein G (2025) Pain prevalence rates and the mediating role of negative affect in adults referred to personality disorder treatment: A cross-sectional study. J Pain 26:104724. 10.1016/j.jpain.2024.104724 39481671 10.1016/j.jpain.2024.104724

[7] van Dooren FEP, Verhey FRJ, Pouwer F, Schalkwijk CG, Sep SJS, Stehouwer CDA, Henry RMA, Dagnelie PC, Schaper NC, van der Kallen CJH, Koster A, Schram MT, Denollet J (2016) Association of Type D personality with increased vulnerability to depression: Is there a role for inflammation or endothelial dysfunction?—The Maastricht Study. J Affect Disord 189:118–125. 10.1016/j.jad.2015.09.028 26433759 10.1016/j.jad.2015.09.028

[8] Garip Y, Güler T, Bozkurt TÖ, Önen S (2020) Type D personality is associated with disease severity and poor quality of life in Turkish patients with fibromyalgia syndrome: A cross-sectional study. Arch Rheumatol 35(1):13–19. 10.5606/ArchRheumatol.2020.7334 32637915 10.5606/ArchRheumatol.2020.7334 PMC7322307

[9] Habra ME et al (2003) Type D personality is related to increased metabolic risk in healthy adults. Psychosom Med 65(1):103–109

[10] Ibrahim ME, Weber K, Courvoisier DS, Genevay S (2020) Big Five Personality Traits and Disabling Chronic Low Back Pain: Association with Fear-Avoidance, Anxious and Depressive Moods. JPR 13:745–754. 10.2147/JPR.S237522 10.2147/JPR.S237522 PMC7167306 32346307

[11] Kaczmarek M (2015) Type D personality and chronic pain: systematic review. Curr Pain Headache Rep 19(10):5026325482

[12] Linton SJ (2000) A review of psychological risk factors in back and neck pain. Spine 25(9):1148–1156 10788861 10.1097/00007632-200005010-00017

[13] Mols F, Denollet J (2010) Type D personality among noncardiovascular patient populations: a systematic review. Gen Hosp Psychiatry 32(1):66–72 20114130 10.1016/j.genhosppsych.2009.09.010

[14] Quirk SE, Koivumaa-Honkanen H, Kavanagh BE, Honkanen RJ, Heikkinen J, Williams LJ (2023) Exploring the comorbidity between personality and musculoskeletal disorders among adults: A scoping review. Front Psychiatry 13:1079106. 10.3389/fpsyt.2022.1079106 36819943 10.3389/fpsyt.2022.1079106 PMC9932280

[15] Sánchez-Díaz M, Montero-Vílchez T, Quiñones-Vico MI, Sierra-Sánchez Á, Ubago-Rodríguez A, Sanabria-de la R, Molina-Leyva A, Arias-Santiago S (2023) Type D Personality as a Marker of Poorer Quality of Life and Mood Status Disturbances in Patients with Skin Diseases: A Systematic Review. Acta Derm Venereol 103:adv846. 10.2340/actadv.v103.2741 36625209 10.2340/actadv.v103.2741 PMC9885290

[16] Schilling R et al (2018) Typ‑D Persönlichkeit bei orthopädischen Patienten. Schmerz 32(4):283–28929987513

[17] Sheng J, Liu S, Wang Y, Cui R, Zhang X (2017) The Link between Depression and Chronic Pain: Neural Mechanisms in the Brain. Neural Plast 2017:9724371. 10.1155/2017/9724371 28706741 10.1155/2017/9724371 PMC5494581

[18] van Middendorp H, Kool MB, van Beugen S, Denollet J, Lumley MA, Geenen R (2016) Prevalence and relevance of Type D personality in fibromyalgia. Gen Hosp Psychiatry 39:66–72. 10.1016/j.genhosppsych.2015.11.006 26804772 10.1016/j.genhosppsych.2015.11.006

[19] Vlaeyen JW, Linton SJ (2000) Fear-avoidance and its consequences in musculoskeletal pain. Pain 85(3):317–332 10781906 10.1016/S0304-3959(99)00242-0

[20] Waddell G, Burton AK (2004) Concepts of rehabilitation for the management of common health problems. TSO, London

[21] Williams L et al (2018) Type D personality and chronic pain: a meta-analysis. J Psychosom Res 110:80–89

[22] Yarat İT (2024) Effects of Type D Personality on SARS-CoV-2-Related Fears, Anxiety, and Depression in Patients with Chronic Pain. Bagcilar Med Bull 9(3):167–174. 10.4274/BMB.galenos.2024.2023-11-103

